# Client/patient perceptions of achieving equity in primary health care: a mixed methods study

**DOI:** 10.1186/s12939-015-0196-5

**Published:** 2015-08-12

**Authors:** Sharareh Akhavan, Per Tillgren

**Affiliations:** Public Health, School of Health, Care and Social Welfare, Mälardalen University, P.O. Box 883, Västerås, 72123 Sweden

## Abstract

**Introduction:**

To provide health care on equal terms has become a challenge for the health system. As the front line in health services, primary care has a key role to play in developing equitable health care, responsive to the needs of different population groups. Reducing inequalities in care has been a central and recurring theme in Swedish health reforms. The aim of this study is to describe and assess client/patient experiences and perceptions of care in four primary health care units (PHCUs) involved in Sweden’s national Care on Equal Terms project.

**Methods:**

Mixed Method Research (MMR) was chosen to describe and assess client/patient experiences and perceptions of health care with regard to equity. There was a focus group discussion, and individual interviews with 21 clients/patients and three representatives of patient associations. Data from the Swedish National Patient Survey (NPS), conducted in 2011 and followed up in 2013, were also used.

**Results:**

The interview data were divided into two main categories and three subcategories. The first category “Perception of equitable health care” had two subcategories, namely “Health care providers’ perceptions” and “Fairness and participation”. The second category “To achieve more equitable health care” had four subcategories: “Encounter”, “Access”, “Interpreters and bilingual/diverse health care providers” and “Time pressure and continuity”. Results from the NPS showed that two of the PHCUs improved in some aspects of patient perceived quality of care (PPQC) while two were not so successful.

**Conclusions:**

Clients/patients perceived health care providers’ perceptions of their ethnic origin and mental health status as important for equitable health care. Discriminatory perceptions may lead to those in need of care refraining from seeking it. More equitable care means longer consultations, better accessibility in terms of longer opening hours, and ways of communicating other than just via voice mail. It also involves continuity in care and access to an interpreter if needed. Employing bilingual/diverse kinds of health providers is a way of providing more equitable primary health care.

## Introduction

The goal of the Swedish health care system is to provide good care on equal terms to all [1, 2]. Health care in Sweden is a public responsibility, financed primarily through taxes that are levied locally by county councils and municipalities. However, during the past decade, several reports from different governmental agencies, and also the Swedish Association of Local Authorities and Regions (SALAR), have revealed that health care is uneven in quality, lacking in accessibility, and not offered on equal terms to all people [3–5]. Annual public health reports from 2013 and 2014 [6, 7] show that inequalities in health in the population have increased, according, for example, to educational level, gender, and country of birth.

To provide health care on equal terms has become a challenge for health care as a whole, and for primary health care in particular. In general, the central attributes of primary care are: being the place of first contact (accessibility), continuity (personal-focused preventive and curative care over time), patient-oriented comprehensiveness, and coordination (including navigation toward secondary and tertiary care) [8]. Primary health care teams provide continuous care for all patient groups, irrespective of social class, gender, ethnicity, etc., and have an important role to play in the provision of equitable care [9]. In Sweden, people are encouraged to visit primary care facilities to meet their medical requirements in the first instance. According to Swedish health care legislation, primary health care is responsible for the population’s needs for basic medical treatment, care, prevention and rehabilitation that do not demand hospital or technical resources, or other special skills [2]. Primary care in Sweden is carried out at nearly 1200 primary care units and is mainly team based (with physicians, district nurses, nurses, and often also physiotherapists and other health care providers) [10]. What characterizes Swedish primary care in comparison with other countries is that a high proportion of care (about 60 %) is provided by the public sector (county councils), and the rest (40 %) by various operations in the private sector [11]. The average number of patients’ visits to physicians in primary care is three per year, which is lower than in the other Nordic countries, while the average in the OECD is twice as high [12]. The Swedish health system is primarily based on universal health care insurance where patients pay a fee varying between 15 and 30 US Dollars per treatment. Since it lies on the front line of health care services, primary health care has a key role to play in developing an equitable health service, responsive to the needs of different population groups. Reducing inequalities in health care and improving the quality of primary care by reducing unacceptable variations in provision have been central and recurring themes in government-initiated health reforms in Sweden. Providing professional care and reducing barriers to access are keys to improving quality in primary health care. In order to apply methods for reducing health inequalities and achieving equity in care, the Swedish government reached an agreement with SALAR to develop and implement a project entitled Care on Equal Terms in disadvantaged areas in June 2011. The project began in the late fall of 2011 and was completed in early 2014 at seven Primary Health Care Units (PHCUs) from five counties/regions in Sweden. In this project, the health care providers, by using so-called Breakthrough methodology, learned about more equitable care and the models developed in their own PHCUs to achieve it.

Client/patient evaluations provide an important way of measuring aspects of primary health care quality. The current study describes and assesses client/patient experiences and perceptions of primary health care when the Care on Equal Terms project was at its concluding phase.

## Methods

Mixed method research (MMR) was chosen to describe and assess client/patient experiences and perceptions of health care. MMR is a type of research in which researchers, especially in the health sciences, combine elements in qualitative and quantitative studies for the purpose of achieving breadth and depth of understanding [13]. MMR involves the intentional collection of both quantitative and qualitative data, and combines the strengths of the two to address specific research questions. This can be achieved by merging qualitative data in the form of texts or images with quantitative data in the form of numeric information. Integration can be achieved by reporting first on the quantitative results and then on quotes or themes that support or refute them (or vice-versa). Then, all the results are reported together, usually in a discussion section [14]. Here, the issues that needed to be described and assessed in MMR were client/patient experiences and perceptions of equitable health. The issues were addressed using a focus group discussion, individual semi-structured interviews, and data from the Swedish National Patient Survey (NPS).

Focus group discussion is a useful vehicle for involving users in issues related to care management and strategy development, needs assessment, participatory planning, and the evaluation of health promotion programs [15–18]. In this study, focus group discussion allowed group participants to agree or disagree with each other, thereby providing insights into the thinking of the group about a range of opinions, experiences and practices related to equitable health care.

The use of semi-structured interviews enables the researcher to prepare a number of questions in advance. The interviewer may also ask spontaneous questions and change the order of the prepared questions as the interview progresses. Semi-structured interviews also permit interviewees to recount their experiences with as little guidance as possible from the interviewer [19]. In this study, semi-structured interviews enabled us to maintain a fairly open framework, which permitted focused, conversational, two-way communication to gain a deeper understanding of the studied phenomena.

The National Patient Survey (NPS), as it related to the four PHCUs investigated in this study, was conducted by SALAR in 2011, with a follow-up in 2013. The NPS gives a recurring national measurement of patient-perceived quality in primary health care, and has been conducted every two years since 2009. The results of the survey are used to develop and improve care from a patient perspective. It should be noted that survey research is often the only means available for developing a representative picture of the perceptions and characteristics of a large population. Properly conducted surveys have three potentially favorable properties: probability sampling, which enables non-biased samples; standardized measurement, which gives comparability between surveys; and the acquisition of special purpose data. Jointly, having these properties meets the needs for data analysis [20].

### Participants

Four of the seven PHCUs that participated in the Care on Equal Terms project were chosen for inclusion in this study. Table 1 illustrates the demographic characteristics of the municipalities in which the chosen PHCUs are located. At municipality level, PHCUs 1 and 3 have a higher density of immigrants than is the case nationally, while PHCUs 1, 2 and 3 have a higher level of attending physicians than other PHCUs nationally [21]. The criteria for a PHCU to be included were: to be located in a disadvantaged area (according to household income and/or the local unemployment rate) and to have planned to develop and implement patient/client-oriented changes within the PHCU. Maximum Variation Sampling [22] was used as a mode of purposive (nonprobability) sampling to cover a broad spectrum of perspectives on the phenomena we were investigating.

**Table 1:** Demographic information on the four studied PHCU and respondents to the NPS in 2011 and adjusted response rates for 2011 and 2013 in the four PHCUs

PHCU	Unemployment level (%)– age 16–64 in 2012	Immigrants (%)	Gini – coefficient	Number of visits to PHCU in 2011^d^	Sex of respondents to the NPS (%)	Mother tongue of respondents to the NPS (%)	Education of respondents to the NPS (%)	Adjusted response rate (%)
	In municipality level^a^	In municipality level^b^	In municipality level ^c^					
		In area level						
PHCU 1– Where interviews with patients/clients were conducted	9.0	27	F 0.36	35591	F 63	Swedish 62	Basic 37	2011 37.2
		36	M 0.38		M 37	Other 38	Upper-secondary 45	2013
							Post-upper-secondary 18	45.3
PHCU 2 – Where interviews with patients/clients were conducted	7.8	19	F 0.33	24727	F 60	Swedish 50	Basic 33	2011
								38.0
		31	M 0.34		M 40	Other 50	Upper-secondary 36	2013
							Post-upper-secondary 31	44.8
PHCU 3 – Where interviews with patient associations were conducted	10.2	28	F 0.34	38473	F 65	Swedish 65	Basic 37	2011
								45.2
		34	M 0.36		M 35	Other 35	Upper-secondary 40	2013
							Post-upper-secondary 23	45.9
PHCU 4 – Where focus group discussion were conducted	7	14	F 0.31	35707	F 58	Swedish 93	Basic 45	2011
								53.4
		31	M 0.31		M 42	Other 7	Upper-secondary 42	2013
							Post-upper-secondary 13	52.9

^a^National level: 6.3

^b^National level: 20

^c^National level: Female: 0.34 - Male: 0.36

^d^National level: 25600

The individuals recruited for interview were clients of the PHCUs that had developed and implemented patient/client-oriented changes. Accordingly, knowledge of their experiences and perceptions would help achieve the aim of the study. The heads of the four PHCUs assisted the research team in recruiting participants by allowing members of the team to be in their PHCU for one week to introduce themselves and the aim of the research to their clients/patients. Those who were interested were interviewed on PHCU premises, in a quiet and closed room. The interview sample (Table 2) came from:

**Table 2:** Socio-demographic information about participants in the semi-structured and focus group interviews

Sex distribution	N (21)
Women	72 %
Men	28 %
Civil status	
Married	66 %
Single with or without children	34 %
Distribution of age	Mean (SD) 36.2 (10.2)
Education	
Lack of basic education	24 %
Basic education	21 %
Upper-secondary	30 %
Post-upper-secondary	25 %
Country of origin	
Sweden	29 %
Other countries	71 %
Number of visits to PHCU per year	
Very often	29 %
Often	24 %
Sometimes	28 %
Seldom	19 %

- A focus group discussion with five Somali mothers who had participated in a newly started Mother Group at one of the studied PHCUs (PHCU 4). They were members of the largest patient group in PHCU 4, and were informed about the study and recruited through their PHCU. The discussion was conducted in a quiet and closed room in the PHCU.
- Individual interviews with 16 clients/patients (both native Swedes and immigrants) at two of the studied PHCUs. They were recruited at random when visiting their PHCU (PHCU 1 or 2), and interviewed in a quiet and closed room on PHCU premises.
- Individual interviews with representatives of three patient associations: the Neuro-patients’ Association (with about 500 members), the Organization for Mentally Disordered Clients (which has contact with 120–130 clients with mental ill-health), and the Anxiety Disorders Society (a national organization with about 1500 members). They were informed about the study and recruited through one of the studied PHCUs (PHCU 3). Two of the interviews were conducted on the associations’ premises, and one at Mälardalen University, in quiet and closed rooms.

### The NPS sample

Table 1 shows the number of questionnaires returned in relation to how many were sent out. The questionnaires were sent to people who had visited their PHCU during the last six months. The adjusted response rate is the number of received forms in relation to those sent out after omitting the people who, for various reasons, had not been able to respond. The background characteristics of the participants in 2011 are shown in Table 1. In 2013 and 2011, when 260 000 questionnaires were sent out nationally each year, the adjusted response rates were 52.7 and 54.0 %, respectively.

### Data collection

Both the focus group discussion and the individual semi-structured interviews were conducted with clients/patients by a Swedish-Somali public health research assistant in Swedish and Somali. Individual semi-structured interviews with representatives of the patient organizations were conducted by the first author in Swedish. They continued until saturation [22] was reached, that is, the point at which no further substantive information was being obtained. The focus group discussion with the Mother Group^1^ and the interviews with the patient organizations were planned group activities within the targeted PHCUs. An interpreter was hired for interviews in Arabic. The focus group discussion lasted 60 min, and the semi-structured individual interviews between 40 and 60 min. Audio recordings were made of all the interviews, which were then transcribed verbatim. The interviews in Somali or Arabic were transcribed in the language of origin, and then translated first to Swedish and then to English. The interviews in Swedish were transcribed and then translated to English. Translation from Swedish to English was performed by the first author in collaboration with the research assistant. Interviews with the representatives of patient associations were conducted in Swedish. The questions in the interviews were open-ended in order to obtain spontaneous information relevant to the study. The research questions that were pre-prepared by the authors were: What is equitable health care for you? How do you define it? How can care be more equitable, what are your thoughts and suggestions? Have you perceived any movement toward more equitable care in your PHCU during the last two years?

Basic requirements of this study were that oral and written information should be exchanged with participants and that written consent should be obtained. The interviews were voluntary, and informants were able to terminate their interview without giving any reason. Participants will remain anonymous. The decoded file is stored by the first author in a program that is only accessible with coded log-in. The study was approved by the Ethical Committee in Uppsala (Dnr: 2013/461).

The variables measured in the NPS included client/patient experiences of Patient Perceived Quality of Care (PPQC): Encounter, Participation, Information, Access, Trust, Usefulness, Recommended to other people who need care, and give an Impression of Quality (of the unit). The survey is conducted in Swedish and six additional languages (used, in total, by about 5 % of the population).

### Data analysis

The data collected from the focus group discussion and individual semi-structured interviews were subjected to content analysis. The analysis was performed by identifying, coding and categorizing the primary patterns that emerged from the data [23, 24]. At a first phase, we read through the transcripts of the interviews and of the focus group discussion several times to get a sense of the material as a whole. At a second phase, sentences or phrases that contained information relevant to the research questions were selected. A third phase followed, which consisted in the systematic analysis of responses in order to code, cut out and sort them according to the aim of the study. The researchers were able to subsume several codes into broad sub-categories. A thorough comparative analysis of these sub-categories resulted in a final multi-category coding structure [25, 26].

The data collected from the NPS were analyzed using SPSS. We compared responses from the NPS in the four studied PHCUs with national averages in order to obtain points of reference. The background variables, to which we had access only for 2013, were: gender, mother tongue (Swedish – yes or no), education, general health status, and number of contacts with health care in the last 6 months.

## Results

Two main categories and three subcategories emerged from the interview data. The first category “Perception of equitable health care” had two subcategories, namely “Health care providers’ perceptions” and “Fairness and participation”. The second category “To achieve more equitable health care” had four subcategories: “Encounter”, “Access”, “Interpreters and bilingual/diverse health care providers”, and “Time duration and continuity”.

### Perceptions of equitable health care

#### Health care providers’ perceptions

The interviews show that health care providers’ attitudes toward foreign-born clients’/patients’ ethnic origin are perceived as a part of equitable health care. One member of the focus group discussion said: “For me, it (equitable health care) means fair treatment … sometimes I wonder if it is due to a lack of language that they treat people this way (FG 1 - PHCU 4)”. Some considered that inequality in care may arise because of language difficulties, while others expressed the view that it is not about language alone: “Sometimes you have a person who can speak the language, yet is treated differently … it can be both language difficulties and also the staff’s perceptions of us (FG 2 - PHCU 4)”. One added: “Be treated like a person and not become fixated on people’s ethnic origin … well … they see Somalis and they see our difficulties in speaking Swedish (FG 3 - PHCU 4)”.

Another interviewed client stated: “… To treat people equally … we should be treated as they treat the Swedes, the same (medical) treatment as for the Swedes, so that we feel safe. But if you are treated differently and don’t feel motivated to visit a PHCU, then there is no point in seeking care. It may be that you’re in a lot of trouble, but decide just to keep it at home (IP 6- PHCU 2)”.

One of the interviewees said: “Equality of care for me is to be listened to ”. She added: “We are from other countries, but we are not stupid, we have enough on our minds (IP 2 - PHCU 1)”.

Health care providers’ perceptions were also discussed by the representative from the Anxiety Disorders Society who stated that equal care meant: “If you come and seek care you want to be treated with respect and to be taken seriously by health care providers … you seek understanding, and expect knowledge about the diseases (IP 1 - PHCU 3)”.

To be listened to and be treated with respect and taken seriously were perceived as important concerns in relation to health care providers’ perceptions.

#### Fairness and participation

According to the interviewees, equity in health care means feeling welcome and being treated like any other citizen. One explained: “Equity in care means that everyone should get a chance to be examined by a doctor. Even when they think it is not needed … you get the chance because sometimes they (health care providers) are also wrong. So everyone should get the same help, there shouldn’t be any differences (FG 1 - PHCU 4).”

Another interviewee, who is illiterate, added: “Help people who are in need. … Explain and provide information in a way that makes sense … for example, with pictures (IP 7 - PHCU 2),” while another expressed the view that equitable care is when: “they (health care providers) do not talk over the heads of patients. It may take time … time should be given to patients so they can say what they want to say (IP 8 - PHCU 2).” Another client added: “Allow patients to be involved in their own care (IP 10 - PHCU 2).”

Another interviewee said that health care would be more equitable if it became fairer, and more non-hierarchic and client-oriented: “They (health care providers) must stop acting as if the health centers are like Fort Knox. They should be accessible to all people, and not just on the phone. It’s just like they get really confused if something happens outside their field of vision. It’s the health care organization that should adapt to the clients/patients, not vice-versa (IP 4 - PHCU 1).”

Fairness and participation in one’s own treatment, and also more non-hierarchic and client-oriented care, were perceived as factors that could be reasonably expected in health care.

### To achieve more equitable health care

#### Encounter

In order to achieve equitable health care, providers should be more aware of the way they encounter patients/clients in different situations. For example, first-time mothers need more support. One of the interviewed mothers in the focus group discussion said: “…we take a very sick child to the PHCU, and they just say (with a nonchalant tone in their voices) ‘take it home’ … they just feel the baby’s skin, and say that it is not dried out yet. They do not help (FG 5 - PHCU 4).” Another added: “… they could at least respond with respect and dignity. When it comes to a child who looks almost lifeless and you are worried, the least they could do is acknowledge your concerns. Look at the child, and then explain why they think you should go back home … (FG 3 - PHCU 4, with mumblings of support from the rest of the group).” According to the interviewees, care would be more equal if “… they listened and took us seriously (FG 2 - PHCU 4).”

Most of the interviewees in PHCUs 1 and 2 felt that during the two years that their PHCU had participated in the Health on Equal Terms project, their health care providers had changed for the better: “The health professional talk in a more friendly way. They smile and you feel welcome (IP 6 - PHCU 2),” another said simply: “They listen more (IP 2 - PHCU 1).”

The Chair of the Neuro-patients’ Association said that he had observed changes in recent years within his PHCU, which was part of the project: “… personnel give patients and clients more respect and attention.” The representative said: “When I came to the PHCU, the staff talked to my assistant and not to me. It has started to change … Now they talk to me first (IP 2 - PHCU 3).”

To be supported with respect and dignity, and to be seen and treated with empathy, were mentioned as important factors in encounters that lead to more equitable health care.

#### Access

Participants in the interviews from PHCUs 1 and 2 were satisfied with access to care as long as they were able to call and make an appointment: “Everyone gets a time after being booked in.” Positive changes in PHCUs 1 and 2 were the queue number system and the drop-in arrangement. One interviewee said: “I feel better now … it feels that the personnel have more control now”: another: “It’s quicker now in the morning when you call” According to some of the interviewees, a drop-in system makes it easier for people who cannot communicate by voice mail.

Some still had problems with waiting times: “The worst thing is when you call and get the message that they will call you back in 2 to 3 h. How do you cope with waiting so long? (FG 3 - PHCU 4)”.

A representative of the Organization for Mentally Disordered Clients stated that the PHCU (PHCU 3) worked well, that the organization had never had any complaints from members, and that access had improved. However, the representative still regarded the voice mail system as a problem. “Then … it’s this voice mail system, and it’s clear that when you have depression or an anxiety attack, or do not hear what they’re saying on the answering machine … then there may be a misunderstanding. So, we often help our members by calling the PHCU from our association’s premises (IP 1 - PHCU 3).” The representative said that contact and communication with health care is something that must be rolled out smoothly. It should not be necessary for members to come to our organization and ask: “Would you please call them for me? Phone hours and voice mail do not work for people with mental illness (IP 1 - PHCU 3)”.

According to the interviewees, shorter waiting times and more efficient ways of communicating with PHCUs would promote better access to health care.

#### Interpreters and bilingual/diverse health care providers

Access to interpreters was also perceived by some interviewees as a factor in achieving more equitable health care. Some interviewees said that if they visited their PHCU at the weekend it could be difficult to book an interpreter, and it caused irritation on both sides when you could not communicate. Most people think it is good that there are doctors who can speak languages other than Swedish. The interviewees said that it is not good to use family members as interpreters.

Some of the interviewees stated that care would be more equal if health care units employed bilingual/diverse health care providers: “Well, for example, I do not like to shake hands with male doctors but if they reach out their hand, I take it. I do not want to end up in the media (laughs) because I refuse a handshake… it’s good that we now have a Somali doctor in our PHCU … he never stretches out his hand. [I remember] the occasion that I met him the first time. I had problems in the genital area, and when I walked into the room there was a Somali man. I almost died! That it should be a man who would examine me down there, and a Somali man at that (laughs)! Thankfully, he took the initiative and arranged for me to have a female doctor instead. It was he who realized how delicate the situation was. I don’t think a Swedish male doctor would have had the same awareness (IP 5 - PHCU 1)”.

#### Time pressure and continuity

Another factor that the participants pointed to as affecting equitable care was the length of an appointment with a nurse or physician: “It’s far too short. More time is needed, interpreting takes time … So that you can have time to say what you want to say (FG 5 - PHCU 4).” Another confirms this, adding. “That’s true. Time is too short … I often leave feeling dissatisfied (FG 3 - PHCU 4)”.

A client who had come to a PHCU with her sick son, who had asthma, said that she was pleased with access to her PHCU, but not satisfied with the continuity of care. It would have been better to be able to meet the same nurse/physician: “The nurse does not know who I am, knows nothing about my son’s situation, and has no time to listen to me (IP 8 - PHCU 2)”.

The representatives of patient organizations were also dissatisfied with continuity of care. One said that members of his association did not like meeting new physicians all the time: “Every time they go to their PHCU, they may have a new doctor … and then the doctor must read about them in the journal. My members want to have the same doctor … but, unfortunately, there are so many around, and there is so much variation (IP 1 - PHCU 3).” A representative of the Organization for Mentally Disordered Clients said: “What is still a big problem is the need always to meet a new doctor (IP 1 - PHCU 3).”

More time for visits to the PHCU and continuity in care were mentioned as crucial factors in achieving more equitable health care.

### The national patient survey

Results from the survey show that in the two PHCUs where interviews with clients/patients were conducted, improvements were made to achieve more equitable care in some aspects of PPQC. They improved Participation, Information, Access and Trust. PHCU 1 scored the same in terms of Encounter and Usefulness, but lower in terms of Overall Impression and higher in terms of being Recommended in 2013 compared with 2011. Compared with the national average, the PHCU was higher in Encounter, Participation, Information, Access and Trust, but lower in Usefulness, being Recommended and Overall Impression in 2013. PHCU 2 improved regarding Participation, Information, Access, Trust, being Recommended and Overall Impression in 2013 compared with 2011, but needed to improve in terms of Encounter and Usefulness. Compared with the national average, PHCU 2 was higher on access but lower on all other aspects of PPQC in 2013. PHCU 3, where the interviews with patient associations were conducted, improved on Encounter but had lower scores on all other aspects of PPQC (expect Usefulness, which was at the same level) in 2013 compared with 2011. Compared with the national average, they had lower scores on all aspects of the PPQC in 2013. PHCU 4, where the focus group discussion was conducted, had lower scores on all aspects of PPQC (expect Encounter, which was at the same level) in 2013 compared with 2011. Compared with the national level, it needed to improve on all aspects of PPQC, especially Access (Table 3).

**Table 3:** PPQC at the studied PHCUs in 2011 and 2013 compared with the national average in 2013

	National level 2013 (%)	PHCU 1 2013 (%)	PHCU 1 2011 (%)	PHCU 2 2013 (%)	PHCU 2 2011 (%)	PHCU 3 2013 (%)	PHCU 3 2011 (%)	PHCU 4 2013 (%)	PHCU 4 2011 (%)
Encounter	90	91	91	81	82	89	88	89	89
Participation	79	80	77	65	61	69	72	74	79
Information	77	80	74	71	67	71	73	70	82
Access	79	93	88	80	73	65	71	64	85
Trust	85	88	87	72	70	77	79	80	88
Usefulness	82	79	79	70	72	74	74	72	86
Recommend	83	82	80	61	59	75	78	80	88
Overall impression	72	70	71	60	59	62	66	67	70

## Discussion

Finding out about client/patient perceptions of health care is important for developing and achieving greater equity. In this study, health equity is highlighted through interviews with clients/patients who have been in contact with a PHCU involved in the Care on Equal Terms project, and through Sweden’s National Patient Survey.

The results show that clients/patients regarded health care providers’ perceptions of client/patient ethnic origin and mental health status as important for achieving equitable health care. Further, the interviewees considered fairness and participation as important factors that contribute to equity in health care. In some of the PHCUs involved in the project, interviewees noted changes in the practices that had been adopted to achieve equitable care. However, they expressed the views that getting more equitable access to health care requires the following: access to interpreters if necessary, continuity (that is, being able to meet the same physician on different occasions), more time for consultations, better accessibility in terms of longer opening hours, and ways of communicating with primary care other than voice mail. They also expressed the views that the health care organization should broaden its perspective, and keep up with diversity and differences in the community. It is the health care organization that should adapt to clients/patients and not vice-versa. Introducing a drop-in system and employing bilingual/diverse health providers were two measures used in the project to provide more equitable care, and were appreciated by the interviewees. Drop-in systems help people with difficulties in expressing themselves to communicate via voice mail, and to make a medical appointment. According to previous research, recruiting bilingual/diverse health professionals also helps to provide more equitable care [27, 28]. Results from the NPS show that two of the PHCUs involved have improved in some aspects of PPQC. However, the two other PHCUs have not been so successful in improving their PPQC.

### Health care providers’ perceptions

In line with the results of this study, previous research [29] has shown the importance of health care providers’ perceptions. Health providers’ perceptions of immigrants/foreign-born patients influence health-related behaviors, medical decisions, quality of care, and health outcomes. Encounters of perceived discrimination, including cases of insensitive, unfriendly or ignorant behavior on the part of health care providers, ethnic discrimination, stereotyping, and receipt of inferior care [30–35] were mentioned. Negative perceptions may seriously affect clients’/patients’ perceptions of the provision of health care.

As one of the interviewees put it, feeling intimidated or unwanted due to the perceptions or judgments of providers may lead patients to avoid seeking health care services, which may lead to poorer overall health, lower quality care [36], and lower levels of health care usage within the immigrant and refugee population [37–39]. At the level of provider/patient interaction, discriminatory perceptions among health care providers can lead to misdiagnosis and misuse of interventions, under-diagnosis, under-utilization of treatment and services, heightened levels of distress, and avoidance of the health care system [40].

The results also show that health care providers’ negative perceptions of clients/patients with mental illness may affect the provision of care and, as previous research shows, are related to low rates of help-seeking and poorer quality of physical health care among people with mental illnesses [41]. It has been shown that good communication between primary care and mental health teams is a prerequisite for effective shared care [42, 43].

### Achieving more equitable health care

The results of this study show that Encounter (one of the elements in the patient surveys) is key to achieving equitable health care. The interviewees perceived a process of othering. In recent years, othering practices have been examined in relation to the experiences of numerous groups, including African American women [44], lesbians [45], immigrants [46, 47], and people with disabilities [48]. Significant health consequences associated with othering have been described. Social experiences, such as discrimination and othering, have been shown to have health consequences, such as shorter life expectancy, higher infant mortality, and hypertension [49, 50].

The results of the current study show that access to care is another important aspect of health provision. According to the interviewees, longer opening hours, drop-in systems, and better service in voice mail systems can enhance equitable access to care. It has been shown that there are variations in expectations of and needs for primary health care between and within population groups [51]. In disadvantaged areas, where the PHCUs involved in this study were located, there is a need for greater access. Equal access for equal need requires conditions under which people with equal needs have equal opportunities to access health care (horizontal equity), while those with unequal needs have appropriately unequal opportunities to access care (vertical equity) [52].

The interviewees in this study perceived that access to interpreters and bilingual/diverse health care providers may achieve more equitable care. In line with the results of this study, previous research [28, 53, 54] points to poor quality of verbal communication and cultural and language skills as factors that may contribute to inequality in health care. Employing bilingual health care providers, using qualified interpreters or health navigators [55], and providing written information in different languages are some suggestions for facilitating communication, and increasing patient satisfaction and understanding. Navigators and bilingual health care providers play an integral role in the changing environment of health care delivery by facilitating access to care, as well as by addressing language and cultural barriers. They break through literacy barriers, build trust, reduce fear, and support the improvement of patient-provider communication. In so doing, navigators may help in delivering better quality and more efficient care, and ensuring access to care for all [56]. Lack of diversity in the leadership and workforce of health care organizations leads to structural policies, procedures, and delivery systems that are inappropriately designed or poorly suited to diverse patient populations [57, 58]. In Sweden, the number of bilingual/diverse health care workers does not mirror the population composition of many of its county councils. For example, among physiotherapists, 9 % are of foreign origin; for administrators, the figure is 10 %, and for nurses and assistant nurses 12 and 15 %, respectively. Very few managers have a non-diverse background. However, 34 and 36 % of physicians and dentists, respectively, have different backgrounds because county councils actively recruit physicians from abroad, mainly from Germany and Poland [59].

According to the interviews, continuity of care is an important factor in achieving more equitable health care. Descriptions of PHCUs [60, 61] have shown that they use agency (temporary) staff, particularly physicians. The extent of use of temporary staff ranged between PHCUs, from a few hours of work to approximately 40 % of the work of all the physicians employed. This is a phenomenon that does not contribute to continuity of care, but instead causes patients to visit different physicians. In Norway, where there were problems with non-continuity of care after the introduction of Fastlegeordningen in 2001, the system was changed so that all residents were registered with a fixed family practitioner. According to evaluations of the reform, it has increased accessibility and continuity of care, resulting in increased patient satisfaction. The reform has also meant better economic conditions for specialists in general, which has made medical schooling and the medical profession more attractive [62, 63].

Continuity of care for people with mental ill-health has provoked theoretical debate about what continuity entails and whether it affects patient outcomes [64]. The participants in this study were unambiguous about what continuity meant for them. They were clear about the negative impact of lack of staffing continuity on their care. This should be a key consideration at a time when mental health services are reviewing care delivery.

The results of this study show also that shortage of time for consultation is perceived as a factor in achieving equitable health care. There is evidence that disadvantaged groups may receive shorter consultations, and that certain groups are consistently less satisfied with their care [65].

### The combination of qualitative and quantitative data: results from the National Patient Survey (NPS), focus group discussion and interviews

By merging data [14] from the NPS, focus group discussion and interviews we were able to gain deeper understanding of the research issues. The variables that are measured in the NPS are Patient Perceived Quality of Care (PPQC), which include: Encounter, Participation, Information, Access, Trust, Usefulness, may Recommend to other people who need care, and Impression of Quality (in this unit). As our interview data show, these variables reflect what clients/patients highlighted in their interviews regarding the achievement of more equitable health care. However, some aspects, such as continuity of care and time for consultation, which were mentioned by interviewees in this study, are not included in the NPS. We can see a concordance between the results of the interviews and the NPS. The results of the NPS show that two of the PHCUs improved some aspects of their PPQC, while two were not so successful. The PHCUs that improved their PPQC compared with the national average also had higher scores on some aspects of PPQC, while the PHCUs that did not improve their relative PPQC had lower scores on all aspects of PPQC. Interviewees from PHCUs 1 and 2, in comparison with PHCUs 3 and 4, were more satisfied with these improvements. A further conclusion that can be drawn from the comparison of the interviews and the NPS is that Encounter is an aspect that should be especially considered for improvement. One interpretation of our results is that the methods that PHCUs 3 and 4 chose for change and for the achievement of more equitable care were not sufficient. Another is that their methods may take more time to lead to visible change. The PHCUs that achieved better results adopted procedures like employing a foreign language(Somali) physician, introducing a drop-in system, longer opening hours, and better and quicker communication services on top of a mail-voice system.

### Methodological considerations

The consistency of the results of this study with those of previous studies indicates its reliability. Validity is addressed by considering credibility, transferability and dependability [66]. Credibility was gained by reading the transcribed interviews several times. This repetitive task ensured that the authors acquired a thorough overview of the transcripts, and safeguarded against the common risk of translation distortion [67]. Furthermore, the process of coding was conducted and cross-checked by both the authors. Shenton [66] considers that transferability lies in demonstrating that the findings of one study can be applied to a wider population. Since the findings of the present study are specific to a small number of particular environments, it is difficult to demonstrate that the conclusions are applicable to other situations and populations. However, by referring to previous research and comparing the results of the present study with those of earlier studies, and also by accumulating the findings of studies staged in different settings, we do enable a more inclusive, overall picture to be obtained. Dependability [68] was achieved by open dialogue between the authors throughout the research process so as to maximize reliability and minimize inconsistencies.

One limitation of the study, however, is the limited number of interviews. Yet, by comparing the results of the interviews with the NPS we gained a deeper understanding of client/patient perceptions. Another limitation may be the use of an interpreter for some interviews and the translation of transcribed interviews, which can have resulted in erroneous meanings, or loss of nuance or misunderstanding of the context [69]. According to previous research [70], using the same interpreter in all interviews is important in order to maintain the internal consistency of translation. For this study, we used the same interpreter. However, without the resources to undertake back translations, there is the possibility that subtle differences in language and terminology were missed.

Some potential limitations of the National Patient Survey (NPS) also require consideration. The NPS as a measuring instrument has some shortcomings, especially regarding sampling [20]. The rate of dropout is high. Individuals who do not have Swedish as their mother tongue will not automatically receive an NPS questionnaire in their native language. Instead, they must turn to the company that conducts the survey to get a questionnaire in their own language, and the survey is only available in six languages other than Swedish. There is a potential risk that the coverage of the target population is not adequate since clients/patients who live in disadvantaged areas are more likely to be low-educated, and sufferers of mental illnesses may not participate to the same extent as those who live in advantaged areas and are high-educated and healthy. As Table 2 shows, the percentage of participants who had Swedish as their mother tongue and a secondary or upper-secondary education was higher than that of clients/patients with a non-Swedish mother tongue (except in one PHCU) and a basic level of education.

It should also be noted that both in the interviews and in the data extracted from the NPS, the longest follow-up time was only two years. In order to be able to follow changes in perceptions and the perceived effects of organizational change, it may be desirable to have a longer time period.

## Conclusions

Clients/patients perceive health care providers’ perceptions of their origin or mental health status as important for providing and achieving equitable health care. Apparent discrimination may lead to those in need of care refraining from seeking it. It also affects client/patient trust in health care providers. Further, fairness and participation are important factors that may contribute to the achievement of equitable health care. According to the results of this study, achieving more equitable care means having more time for consultations, better accessibility in the form of longer opening hours, and ways of communicating with primary care other than voice mail. It also means continuity of care and having access to an interpreter if needed. Employing bilingual/diverse health providers is another way of providing more equitable care.

## Footnotes

1 The Mother Group - A group in one of the PHCUs, in which mothers of Somali background participated and obtained information in areas like infant health. They met once a week for 6 months, with an interpreter to help with communication.

## References

[1] National Board of Health and Welfare: The 2009 Swedish Health Care Report. Västerås: Edita Västra Aros; 2010. http://www.socialstyrelsen.se/Lists/Artikelkatalog/Attachments/8495/2009-126-71.pdf.

[2] Social departement: 2013. Hälso-och sjuvårdslag (1982:763). [Trans: Health and care law].

[3] National Board of Health and welfare. Hälso- och sjukvård – lägesrapporter 2007. Stockholm; 2008. [Trans: Health and health care, Current report for 2007]. http://www.socialstyrelsen.se/Lists/Artikelkatalog/Attachemnets/8864/2008-131-17_20081317_rev2.pd.

[4] National Statistics Central Board. Ohälsa och sjukvård 1980–2005, Levnadsförhållandena; 2006. Rapport nr. 113. [Trans: Ill-health and health care between 1980 and 2005, Life conditions].

[5] The Swedish Association of Local Authorities and Regions. Sveriges kommuner och landsting. (2009). Vård på (o)lika villkor. En kunskapsöversikt om sociala skillnader i svensk hälso- och sjulvård. Stockholm: Sveriges kommuner och landsting. [Trans: Health care in equal terms. A knowledge overview on social differences in Swedish health care].

[6] National Board of Health and Welfare & National Institute of Public Health. Folkhälsan I Sverige. Årsrapport 2013. [Trans: Public health in Sweden, Yearly report].

[7] National Board of Public Health. Folkhälsan I Sverige. Årsrapport 2014. [Trans: Public health in Sweden, Yearly report].

[8] Starfield B (1994). Is primary care essential?. The Lancet.

[9] De Maeseneer J, Willems S, De Sutter A, Van de Geuchte I, Billings M (2007). Primary health care as a strategy for achieving equitable care: a literature review commissioned by the Health Systems Knowledge Network.

[10] Björkelund C, Maun A. European Forum for Primary Care. Stockholm; April 2015. http://www.euprimarycare.org/column/primary-care-sweden

[11] National Board of Health and Welfare. Tillståndet och utvecklingen inom hälso-och sjukvård och socialtjänst. Lägesrapport. Stockholm; 2014. [Trans: Situation and development of health care and social work. Yearly report].

[12] Pettersson S. Kostnader och produktion i primärvårdens vårdval. Uppföljning av vårdvalets primärvård som en del av den samlade hälso- och sjukvården. Stockholm, Sveriges läkarförbund, Stockholm; 2014. [Trans: The costs and production in free choice in primary health care. Follow-up of free choice in primary health care as part of the organization. Report from the Physicians association]. https://www.slf.se/upload/Lakarforbundet/primarvardenskostnaderapril2014.pdf

[13] Taylor B. Mixed methods research. In: Qualitative reserach in the helath sciences- Methodologies, methods and processes. Ed. Taylor B, Francis K. Newyork: Routledge; 2013:162-176.

[14] Meissner HI. Best practices for mixed methods research in the health sciences. National Institutes of Health. Office of Behavioral and Social Sciences Research. http://obssr.od.nih.gov/scientific_areas/methodology/mixed_methods_research/section2.aspx#What is mixed methods research

[15] Gregory S, McKie L (1991). The smear test: listening to women’s views. Nursing Standard.

[16] Richardson CA, Rabiee F (2001). ‘A Question of Access’ – an exploration of the factors influencing the health of young males aged 15–19 living in Corby and their use of health care services. Health Education Journal.

[17] Kitzinger J (1995). Qualitative research: introducing focus groups. British Medical Journal.

[18] Higingbottom G (1998). Focus groups: their use in health promotion research. Community Practitioner.

[19] Morse JM, Field PA (1996). Nursing research – the application of qualitative approaches.

[20] Floyd Jr, Flower J. Survey RESEARCH. Applied social research methods series, No. 1. Sage Publication; 2009.

[21] Swedish Association of Local Authorities and Regions (SALAR). Vård på lika villkor- ett Lärande projekt, Primärvårdens förutsättningar och befolkningens vårdbehov. EO: SALAR; 2014. [Trans: Health and care on equal terms – A learning project on primary health care conditions and population need].

[22] Lisa M (2008). The sage encyclopedia of qualitative research methods.

[23] Krippendorf K (1980). Content analysis: An introduction to its methodology.

[24] Patton MQ (1990). Qualitative evaluation and research method.

[25] Holme IM, Solvang BK (1991). Forskningsmetodik - Om kvalitativa och kvantitativa metoder. Research methodology – about qualitative and quantitative methods.

[26] Graneheim UH, Lundman B (2004). Qualitative content analysis in nursing research: concepts, procedures and measures to achieve trustworthiness. Nurse Education Today.

[27] Akhavan S, Karlsen S (2013). Practitioner and client explanations for ethnic disparities in Swedish health care - a qualitative study. Journal of Immigrant and Minority Health.

[28] Akhavan S (2012). Midwives’ views on factors that contribute to health care inequalities among immigrants in Sweden: a qualitative study. International Journal for Equity in Health.

[29] Dias S, Gama A, Cargaleiro H, Martins MO (2012). Health workers’ perceptions toward immigrant patients: a cross-sectional survey in primary health care services. Hum Resour Health.

[30] Access Alliance (2005). Racialized groups and health status: a literature review exploring poverty, housing, race-based discrimination and access to health care as determinants of health for racialized groups.

[31] Beiser M, Noh S, Hou F, Kaspar V, Rummens J (2001). Southeast Asian refugees’ perceptions of racial discrimination in Canada. Canadian Ethnic Studies.

[32] Johnson JL, Bottorf JL, Browne AJ, Grewal J, Hilton BA, Clarke H (2004). Othering and being othered in the context of health care services. Health Communication.

[33] Noh S, Kaspar V, Wickrama KAS (2007). Overt and subtle racial discrimination and mental health: Preliminary findings for Korean immigrants. American Journal of Public Health.

[34] Stewart D, Gagnon AJ, Dougherty G, Saucier JF, Wahoush O (2008). Postpartum depression symptoms in newcomers. Canadian Journal of Psychiatry.

[35] Wahoush O (2009). Equitable health-care access: the experiences of refugees and refugee claimant mothers with an ill preschooler. Canadian Journal of Nursing Research.

[36] Elliott SJ, Gillie J (1998). Moving experiences: a qualitative analysis of health and migration. Health Place.

[37] Pérez CE. Health status and health behavior among immigrants. Supplement to Health Reports, Statistics Canada, Catalogue 82-003-SIE, 1–12, Ottawa, ON 2002. Retrieved from http://www.statcan.gc.ca/pub/82-003-s/2002001/pdf/82-003-s2002005-eng.pdf

[38] Statistic Central Board. Ohälsa och sjukvård 1980–2005, Levnadsförhållandena; 2006. Rapport nr. 113. [Trans: Ill-health and health care between 1980 and 2005, Life conditions].

[39] Akhavan S: The health and working conditions of female immigrants in Sweden, PHD thesis. Karolinska Institute: Public Health Department; 2006.

[40] Pollock G, Newbold BK, Lafrenière G, Edge S (2012). Discrimination in the Doctor’s Office: Immigrants and Refugee Experiences. Critical Social Work.

[41] Thornicroft G, Rose D, Kassam A (2007). Discrimination in health care against people with mental illness. International Review of Psychiatry.

[42] Bindman J, Johnson S, Wright S, Szmukler G, Bebbington P, Kuipers E (1997). Integration between primary and secondary services in the care of the severely mentally ill: patients’ and general practitioners’ views. Br J Psychiatry.

[43] Gask L, Sibbald B, Creed F (1997). Evaluating models of working at the interface between mental health services and primary care. Br J Psychiatry.

[44] Taylor JY (1999). Colonizing images and diagnostic labels: oppressive mechanisms for African American women’s health. Advances in Nursing Science.

[45] Hall JM, Stevens PE, Meleis A (1994). Marginalization: a guiding concept for valuing diversity in nursing knowledge development. Advances in Nursing Science.

[46] Anderson J, Reimer Kirkham S, Strong-Boag V, Grace S, Eisenberg A, Anderson J (1998). Constructing nation: the gendering and racializing of the Canadian health care system. Painting the maple: essays on race, gender, and the construction of Canada.

[47] Akhavan S. Class, gender and ethnicity in relation to health care – A study on social disparities in the Swedish health care. In: Reconsidering social identification - race, gender, class and caste. JanMohamed A (Eds.). Routledge; 2011: 376–400.

[48] Wendell S (1996). The rejected body.

[49] Krieger N. Embodying inequality: a review of concepts, measures, and methods for studying health consequences of discrimination. International Journal of Health Services. London: New Delhi; 1999;29:295–352. 10.2190/M11W-VWXE-KQM9-G97Q 10379455

[50] Krieger N, Sidney S (1996). Racial discrimination and blood pressure: the CARDIA Study of young Black and White adults. American Journal of Public Health.

[51] Campbell JL, Ramsay J, Green J (2001). Age, gender, socioeconomic, and ethnic differences in patients’ assessments of primary health care. Quality in Health Care.

[52] Oliver A, Mossialos E (2004). Equity of access to health care: outlining the foundations for action. J Epidemiol Community Health.

[53] Hjärn A, Haglund B, Persson G, Rosen M (2001). Is there equity in access to health services for ethnic minorities in Sweden?. European Journal of Public Health.

[54] Helman CG (1994). Culture, health and illness.

[55] Nguyen TU, Tran JH, Kagawa-Singer M, Foo MA (2011). A qualitative assessment of community-based breast health navigation services for Southeast Asian women in Southern California: recommendations for developing a navigator training curriculum. Am J Public Health.

[56] Natale-Pereira A, Enard KR, Nevarez L, Lovell A, Jones LA. The role of patient navigators in eliminating health disparities. Supplement: National Patient Navigation Leadership Summit (NPNLS): Measuring the Impact and Potential of Patient Navigation, Supplement to Cancer 2011, August, 117(15):3541–3550. 10.1002/cncr.26350 21780086

[57] Evans RM (1999). Increasing minority representation in health care management. Health Forum J.

[58] Nickens HW (1992). The rationale for minority-targeted programs in medicine in the 1990s. JAMA.

[59] TCO, Arbetsvärlden. 2014. [The Swedish Confederation for Professional Employees newspaper, The working life].http://www.arbetsvarlden.se/fa-med-utlandskbakgrund-i-varden/

[60] Gustaffson G, Aytar O, Akhavan S, Bogg L, Söderlund A, Tillgren P. Områdesbeskrivning av sju vårdverksamheter : Primärvårdens förutsättningar och befolkningens vårdbehov. Stockholm: EO; 2014. [Trans: Area description of seven health care operations: primary care conditions and the population's health needs]. http://mdh.diva-portal.org/smash/get/diva2:726723/FULLTEXT01.pdf

[61] Akhavan S, Aytar O, Bogg L, Söderlund A, Tillgren P. Blev det genombrott? Västerås: Mälardalen Univeristy Press; 2014. [Trans: Did we had a breakthrough?]. http://www.diva-portal.org/smash/get/diva2:729960/FULLTEXT01.pdf

[62] Stolt M. Fastelegeordning i Norge har gett primärvården 700 nya läkare på två år. Läkartidningen. 2003;100(40):31–3. [Trans: Fast Medical Scheme in Norway gave 700 new physicians in two years]. 14579671

[63] Hetlevik Q, Gjesdal G (2012). Personal continuity of care in Norwegian general practice: a national cross-sectional study. Scandinavian Journal of Primary Health Care.

[64] Johnson S, Prosser D, Bindman J, Smukler G (1997). Continuity of care for the severely mentally ill: concepts and measures. Soc Psychiatry Psychiatr Epidemiol.

[65] Harris MF, Harris E, Roland M (2004). Access to primary health care: three challenges to equity. Australian Journal of Primary Health.

[66] Shenton AK (2004). Strategies for ensuring trustworthiness in qualitative research projects. Education for Information.

[67] Squires A (2008). Language barriers and qualitative nursing research: methodological considerations. International Nursing Review.

[68] Polit DF, Beck CT (2011). Nursing research: generating and assessing evidence for nursing practice.

[69] Pitchforth E, van Teijlingen E (2005). International public health research involving interpreters: a case study from Bangladesh. BMC Public Health.

[70] Lopez GI, Figueroa M, Connor SE, Maliski SL (2008). Translation barriers in conducting qualitative research with Spanish speakers. Qualitative Health Research.

